# For Horticulturists and Amateurs

**Published:** 1869-11

**Authors:** 


### FOR HORTICULTURISTS AND AMATEURS.

An old but very useful and convenient kind of flower-pot is reported among the French patents.

These pots are made of wire-gauze, of perforated tin, or galvanized sheet-iron. These pots are placed within another pot made of china or glazed ware: the space between the outer and inner pots is filled with earth.

Should the plants be set out in the open ground, the gauze-pot is taken from the ornamental vase and placed in the soil; the roots communicate through the openings of the perforated pot with the outer soil, and derive nourishment from it. When winter approaches, the pots are taken up and again placed in ornamental or other pots.

By the use of this simple contrivance, many valuable plants which require to be housed during the winter might be saved, which are now lost by disturbing the roots.

